# The histone demethylase JMJD2C constitutes a novel NFE2 target gene that is required for the survival of JAK2^V617F^ mutated cells

**DOI:** 10.1038/s41375-023-01826-y

**Published:** 2023-01-28

**Authors:** Anne Marie Staehle, Jan Caspar Peeken, Georg Vladimirov, Mirjam Elisabeth Hoeness, Sarolta Bojtine Kovacs, Nikolaos Karantzelis, Albert Gruender, Christoph Koellerer, Jonas Samuel Jutzi, Heike Luise Pahl, Hans Felix Staehle

**Affiliations:** 1 Division of Molecular Hematology, University Medical Center Freiburg, Faculty of Medicine, University of Freiburg, Freiburg im Breisgau, Germany; 2 Department of Radiation Oncology, Klinikum rechts der Isar, Technical University of Munich (TUM), Munich, Germany; 3 Division of Hematology, Department of Medicine, Brigham and Women’s Hospital, Harvard Medical School, Boston, MA 02115 USA

**Keywords:** Translational research, Preclinical research, Myeloproliferative disease

## Abstract

The transcription factor NFE2 is overexpressed in most patients with myeloproliferative neoplasms (MPN). Moreover, mutations in NFE2, found in a subset of MPN patients, strongly predispose for transformation to acute leukemia. Transgenic mice overexpressing NFE2 as well as mice harboring NFE2 mutations display an MPN phenotype and spontaneously develop leukemia. However, the molecular mechanisms effecting NFE2-driven leukemic transformation remain incompletely understood. Here we show that the pro-leukemic histone demethylase JMJD2C constitutes a novel NFE2 target gene. JMJD2C expression is elevated in MPN patients as well as in NFE2 transgenic mice. Moreover, we show that loss of JMJD2C selectively impairs proliferation of JAK2^V617F^ mutated cells. Our data suggest that JMJD2C represents a promising drug target in MPN and provide a rationale for further investigation in preclinical and clinical settings.

## Introduction

Despite discovery of the JAK2, CALR and MPL driver mutations, the molecular etiology of Myeloproliferative Neoplasms (MPN) remains incompletely understood. Current pharmacological therapies lack disease-modifying activity, necessitating the development of improved therapeutic strategies. The transcription factor NFE2 has been identified as an important player in MPN pathophysiology. A large majority of MPN patients display increased NFE2 levels [1] and transgenic NFE2 overexpressing mice develop an MPN phenotype with spontaneous transformation to acute leukemia [2]. Moreover, NFE2 mutations found in a subset of MPN patients strongly predispose for leukemic transformation both in patients and in mice [3–5]. As both the molecular mechanisms causing NFE2 dysregulation in MPN as well as its functional consequences are incompletely understood, their investigation may delineate novel targetable pathways in MPN.

In order to elucidate downstream effectors of NFE2 activity, we analyzed the transcriptome of human CD34^+^ hematopoietic stem and progenitor cells (HSPCs) following overexpression or silencing of NFE2 [6]. Subsequently, we combined our gene expression data with published NFE2 chromatin immunoprecipitation sequencing (ChIP-seq) data [7], generating a list of putative NFE2 target genes [8]. Among these potential targets, we have previously confirmed NFE2-driven transcriptional regulation of the proinflammatory cytokine interleukin 8 (IL8) and the histone demethylase JMJD1C [6, 8]. IL8 levels constitute an independent prognostic factor for survival in primary myelofibrosis (PMF) patients [9], while the pro-leukemic JMJD1C represents a promising drug target in myeloid malignancies [10]. However, we have recently shown that JMJD1C is dispensable for JAK2^V617F^-driven myeloproliferation [11]. Nonetheless, other NFE2 target genes remain putative novel drug candidates in MPN.

In addition to IL8 and JMJD1C, we identified the histone demethylase JMJD2C / KDM4C as a potential NFE2 target gene [6–8]. JMJD2C is upregulated in primary acute myeloid leukemia (AML) cells as well as in AML cell lines [10]. JMJD2C depletion in MLL-rearranged leukemia cells increases differentiation, promotes apoptosis and, upon transplantation, prolongs survival of recipient mice [12]. Ernst et al. recently showed that JMJD2C mRNA expression is also elevated in MPN patients. JMJD2C deletion attenuated growth of JAK2^V617F^-positive HEL cells and, upon transplantation into a xenograft model, increased survival of recipient mice [13]. These data suggest that JMJD2C constitutes an attractive novel therapeutic target in myeloid malignancies. Here we show that NFE2 directly regulates JMJD2C expression and provide mechanistic insights into the effects of altering JMJD2C activity.

## Materials and methods

Materials and methods are described in the Supplementary Material.

## Results

We have previously identified a list of 60 epigenetic regulators constituting potential NFE2 target genes by interrogating NFE2 ChIP-seq data in K562 cells [7]. Amongst these, the histone demethylase *JMJD2C* ranked highly and constitutes an attractive target due to its pro-leukemic role in myeloid malignancies [10]. We therefore investigated whether JMJD2C plays a role in the pathophysiology of Myeloproliferative Neoplasms (MPN).

### The histone demethylase *JMJD2C* is a novel NFE2 target gene

**Fig. 1:**
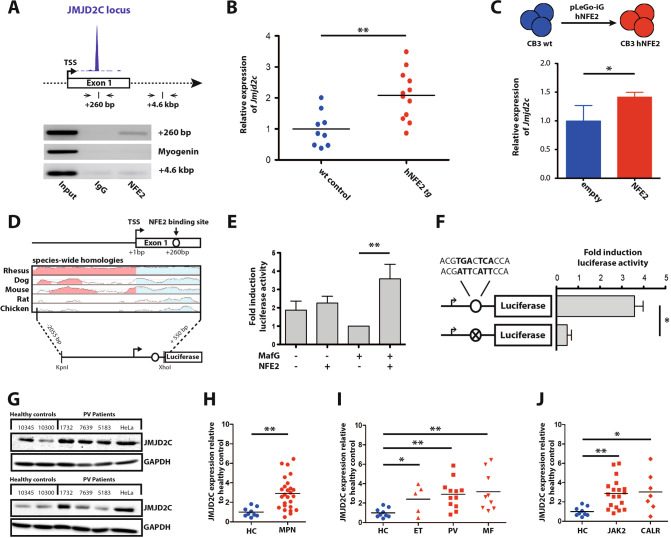
JMJD2C constitutes a novel NFE2 target gene. **A** Top: NFE2 ChIP-seq at the *JMJD2C* locus [7]. Bottom: ChIP PCR of HEL cells using an antibody against NFE2 or an IgG control, as depicted. PCR primers flank the predicted binding site at +260 bp or negative control sites at +4.6 kb of the *JMJD2C* locus and the myogenin locus. **B** *Jmjd2c* mRNA expression in BM of hNFE2tg mice (*n* = 12) or littermate controls (*n* = 9) determined by RT-qPCR. Expression is normalized to *B2m* expression. **C** *Jmjd2c* mRNA levels in CB3 cells following infection with pLeGO-iG-NFE2-wt or an empty control vector. GFP positive cells were sorted and *Jmjd2c* expression measured as described in panel 1B. **D** Illustration of the *JMJD2C* reporter gene construct. A circle indicates the predicted NFE2 binding site +260 bp relative to the transcription start site (TSS). **E** Cotransfection of HEK-293T cells with the *JMJD2C* luciferase reporter gene construct and different combinations of expression plasmids encoding NFE2 and MafG cDNAs, as depicted. **F** Disruption of the potential NFE2 binding site by site directed mutagenesis (crossed circle). Altered bases are shown in bold. **E**, **F** Data were normalized to the wt *JMJD2C* reporter gene construct cotransfected with MafG alone, which was set as one. **G** Exemplary western blots depicting JMJD2C expression in lysates from PB granulocytes. GAPDH was used as a loading control. **H** Densitometric analysis of JMJD2C western blots, *n* = 26 MPN patients and *n* = 9 healthy controls. **I** JMJD2C protein expression across different MPN subtypes (ET = 5, PV = 12, PMF = 9). **J** JMJD2C protein expression according to mutational status (JAK2^V617F^ = 19, CALR = 7). **C**, **E**, **F** Data are represented as mean of at least three independent experiments. **B**, **C**, **E**, **F**, **H**–**J** *p < 0.05, **p < 0.01 by Student’s *t* test.

To confirm the NFE2 binding site identified by ChIP-seq at +260 bp of the *JMJD2C* locus ([7], Fig. 1A), we performed ChIP assays in HEL cells. A strong enrichment of NFE2 was observed around the site identified at the *JMJD2C* locus (Fig. 1A). Concomitantly, *Jmjd2c* mRNA expression was significantly elevated in the bone marrow of transgenic mice overexpressing hNFE2 compared to wild-type littermate controls (Fig. 1B). Moreover, lentiviral introduction of hNFE2 into CB3 cells, an erythroid cell line devoid of NFE2 due to viral insertion, was sufficient to induce *Jmjd2c* expression (Fig. 1C). Taken together, these data show that the *JMJD2C* locus is bound by NFE2 and that presence of NFE2 increases JMJD2C expression.

To evaluate whether NFE2 is sufficient to directly transactivate the JMJD2C promoter, we performed luciferase assays. The region of interest, bp −2220 to +522 of the *JMJD2C* locus, was cloned into a luciferase reporter vector (Fig. 1D) and cotransfected into HEK-293T cells together with plasmids expressing the NFE2 cDNA either with or without its binding partner MafG (Fig. 1E). As hypothesized, we observed a significant increase of luciferase activity only in the presence of both NFE2 and MafG. In turn, site directed mutagenesis of the NFE2 recognition sequence led to a significant decrease of signal intensity (Fig. 1F). NFE2 therefore binds the *JMJD2C* locus and mediates its transcriptional activation.

### JMJD2C expression is increased in MPN patients

Since NFE2 is overexpressed in the vast majority of MPN patients [1], we investigated whether increased NFE2 in MPN patients increases JMJD2C protein expression. Peripheral blood granulocytes from MPN patients and healthy controls were analyzed by Western Blots (Fig. 1G–J). Quantification of JMJD2C protein expression from *n* = 26 MPN patients and *n* = 9 healthy controls (HC) revealed significantly elevated JMJD2C protein levels in patients with essential thrombocythemia, polycythemia vera and primary myelofibrosis (Fig. 1H, I). Likewise, *JMJD2C* mRNA levels were significantly elevated in post-MPN AML patients (Supplementary Fig. S1). No significant difference was observed between JAK2^V617F^- and CALR-mutated patients (Fig. 1J). Moreover, mRNA expression of *NFE2* and *JMJD2C* correlated significantly (Supplementary Fig. S2), underscoring a possible role for this histone demethylase in the pathophysiology of MPN.

### JMJD2C is required for the survival of JAK2^V617F^ mutated cells

**Fig. 2:**
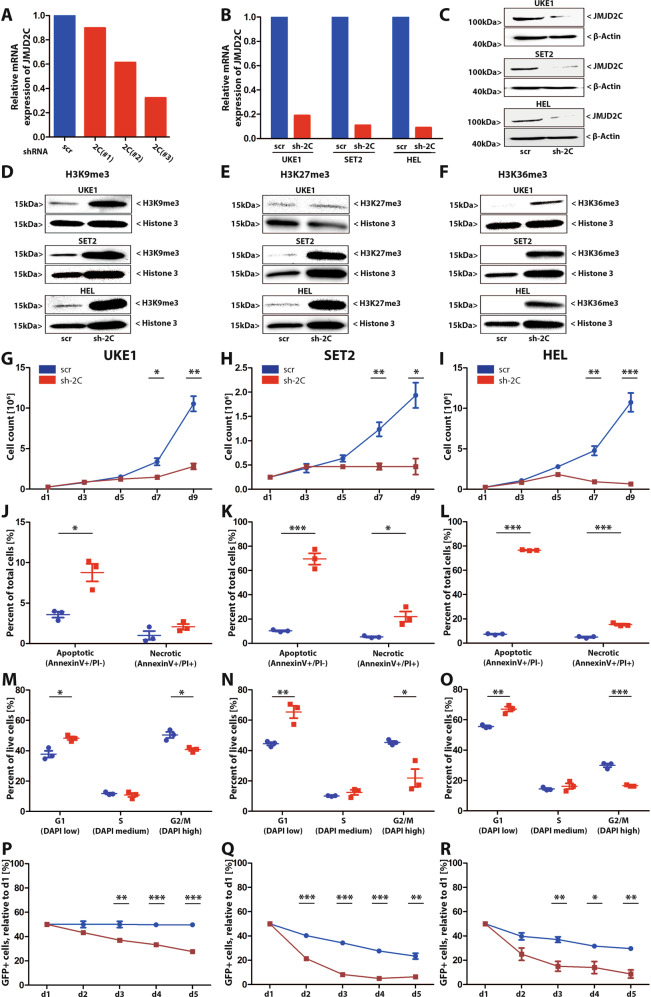
JMJD2C is required for survival of JAK2V617F mutated cells. **A** UKE1 cells were lentivirally transduced with vectors carrying shRNAs against *JMJD2C* (sh-2C #1–3) or a scrambled control (scr). Cells were harvested on day 5 for RNA isolation and subsequent analysis by RT-qPCR. **B**, **C** Validation of sh-2C #3 in UKE1, SET2 and HEL cells by RT-qPCR (**B**) and western blotting (**C**) using an anti-JMJD2C antibody, actin served as a loading control. **D**–**F** Western blotting of JMJD2C-depleted or wt UKE1, SET2 and HEL cells with antibodies against H3K9me^3^ (**D**), H3K27me^3^ (**E**) and H3K36me^3^ (**F**). Histone H3 served as a loading control. **G**–**I** Proliferation of UKE1 (**G**), SET2 (**H**) and HEL (**I**) cells after introduction of JMJD2C-shRNA (sh-2C) or the scrambled (scr) control. For each condition, 0.25 million cells were seeded and infected on day 1. Cells were counted every second day and used for final analysis on day 9. **J**–**L** Detection of apoptotic (AnnexinV+/PI−) and necrotic (AnnexinV+/PI+) cells on day 9 by FACS analysis in UKE1 (**J**), SET2 (**K**) and HEL (**L**) cells. **M**–**O** Cell cycle analysis: detection of cells in G1 (DAPI low), S (DAPI medium) and G2/M (DAPI high) phase on day 9 by FACS analysis in UKE1 (**M**), SET2 (**N**) and HEL (**O**) cells. **P**–**R** Competitive growth of shRNA (sh-2C or scr) infected UKE1 (**P**), SET2 (**Q**) and HEL (**R**) cells. 0.1 million infected (GFP^+^) cells were seeded at a 1:1 ratio with uninfected cells on day 1. The proportion of GFP^+^ cells was determined daily. **G**–**R** Data are represented as mean +/− SEM of three independent experiments. *p < 0.05, **p < 0.01, ***p < 0.001 by Student’s *t* test.

Since JMJD2C deficiency has been shown to attenuate leukemogenicity in AML (reviewed in Staehle et al. 2021), we hypothesized that JMJD2C selectively impacts proliferation of JAK2^V617F^ cells. Hence, we determined the effect of normalizing elevated JMJD2C levels in JAK2^V617F^-positive cells using RNA interference. The most effective shRNA, construct #3 (Fig. 2A) was selected in UKE1 cells, yielding approximately 80–90% reduction in JMJD2C expression, as determined by both RT-qPCR and western blotting (Fig. 2B, C). JMJD2C depletion strongly increased global levels of its target histone marks H3K9me3, H3K27me3 and H3K36me3 (Fig. 2D–F).

JAK2^V617F^-positive UKE1, SET2 and HEL cells were lentivirally transduced with the JMJD2C shRNA or a scrambled control. Transduction efficiencies, determined by FACS analysis on day 5, reached nearly 100 % (Fig. S3). JMJD2C knockdown significantly reduced proliferation of UKE1, SET2 and HEL cells after 7–9 days (Fig. 2G–I). Moreover, Annexin V/PI staining showed an increased proportion of apoptotic and necrotic cells in JMJD2C depleted cells (Fig. 2J–L, Supplementary Fig. S4). Concomitantly, cell cycle analysis revealed reduced proportions of G2/M and increased proportions of G1 phase cells (Fig. 2M–O, Supplementary Fig. S5). In a competitive proliferation assay, we mixed JMJD2C shRNA depleted or scrambled control infected cells with uninfected cells at a 1:1 ratio. While scrambled control infected cells showed no or only a slight reduction in proliferation compared to uninfected cells, the proportion of JMJD2C depleted cells diminished rapidly, indicating that decreased JMJD2C levels confer a proliferative disadvantage (Fig. 2P–R). In contrast, in JAK2^V617F^-negative K562 and U937 cells, JMJD2C depletion did not affect proliferation, apoptosis or cell cycle progression and yielded no competitive disadvantage (Supplementary Fig. S6). Loss of JMJD2C expression therefore selectively impairs proliferation of JAK2^V617F^ mutated cells, at least in part through increased apoptosis and cell cycle arrest.

## Discussion

Elevated NFE2 expression and mutations in NFE2 strongly predispose for transformation to acute leukemia both in MPN patients and in murine models [2–5]. However, NFE2 itself constitutes a difficult drug target, as pharmacological modulation of transcription factors remains challenging. Thus, defining the pathways affected by elevated NFE2 levels may identify more eligible targets for MPN therapy.

Here, we demonstrate that the histone demethylase JMJD2C constitutes a novel NFE2 target gene (Fig. 1). JMJD2C plays a pro-proliferative role in selected myeloid malignancies, however, its activity is context dependent. Loss of JMJD2C does not affect BCR-ABL, AML-ETO and PML-RARA transformed cells (Supplementary Fig. S6, [12]). Conversely, in MLL-driven AML and in JAK2^V617F^-positive MPN, targeting JMJD2C reduces leukemogenicity by increasing differentiation, promoting cell cycle arrest and enhancing apoptosis [10, 12, 13].

As a histone demethylase, JMJD2C affects cell proliferation in myeloid malignancies via epigenetic mechanisms, removing repressive H3K9me^3^, H3K27me^3^ and activating H3K36me^3^ histone marks (Fig. 2D–F, [12, 13]). In MLL-driven AML, elevated JMJD2C levels increase expression of pro-proliferative targets, among them Myc, Hoxa9, and Meis1 [12], while increased JMJD2C activity in JAK2^V617F^-positive HEL cells prevents cellular senescence [13]. JMJD2C depletion thus affects distinct neoplastic pathways in specific clinical entities. In contrast, reduction of JMJD2C levels does not impair healthy hematopoiesis [14], arguing for a therapeutic window in MPN and AML.

To identify changes in gene expression caused by JMJD2C depletion selectively in JAK2^V617F^ cells, we compared RNA-seq data from two cellular models, following JMJD2C targeting: HEL (JAK2^V617F^) and MLL-GAS7 (JAK2 wt) (Supplementary Fig. S7). Genes regulated by HIF1A, JUN and SP1 are selectively enhanced by JMJD2C depletion in JAK2^V617^ positive cells, while Myc signaling and TP53 targets are altered both in JAK2^V617F^ and JAK2 wt cells (Supplementary Fig. S7).

Currently available small molecule JMJD2C inhibitors target the enzyme’s catalytic or reader domain [15]. TACH101, an orally available, reversible, competitive KDM4-family inhibitor, recently entered a phase I/II trial for advanced and metastatic GI tumors (NCT05076552). Safety and tolerability data from this first-in-human trial will determine whether JMJD2C-inhibition is feasible in MPN patients, who carry a lower disease burden. If inhibition is well tolerated, JMJD2C represents a promising drug target in MPN possibly for synergistic use with JAK inhibitors, as Ernst et al. very recently demonstrated that JAK2^V617F^-mutated cells functionally depend on JMJD2C even during exposure to Ruxolitinib [13].

## Supplementary information

Supplemental Material

## Data Availability

Original datasets are available from the corresponding author on reasonable request.
