# Supplementary material for: The histone demethylase JMJD2C constitutes a novel NFE2 target gene that is required for the survival of JAK2V617F mutated cells
Source: Leukemia. 2023 Jan 28;37(4):919–23. doi: 10.1038/s41375-023-01826-y (PMC10079541; doi:10.1038/s41375-023-01826-y)
Supplement: Supplementary file 1 — Supplemental Material [file 41375_2023_1826_MOESM1_ESM.docx]

**Supplemental material**


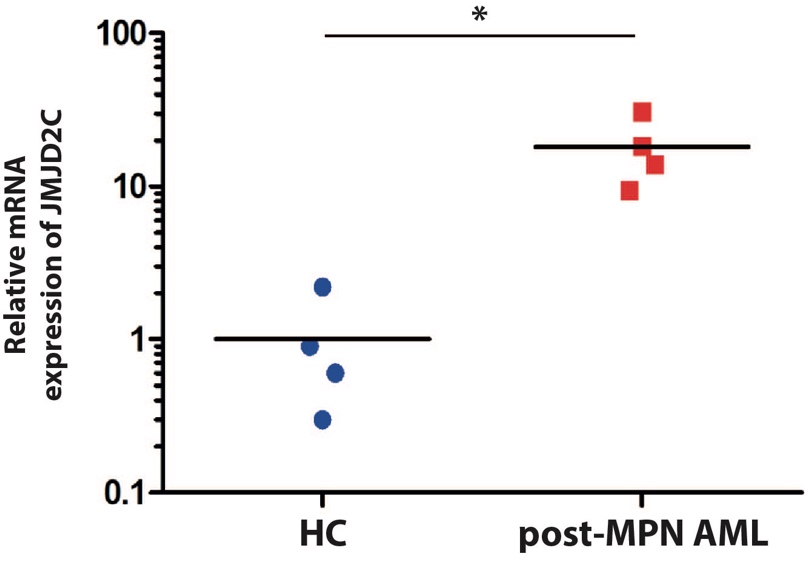


**Figure S1: JMJD2C expression in post-MPN AML patients.** *JMJD2C* mRNA expression in PB samples of n=4 healthy controls (blue) and n=4 post-MPN AML patients (red) by RT-qPCR. Expression is normalized to *B2m* expression. *p<0.05 by Student’s t test.

**
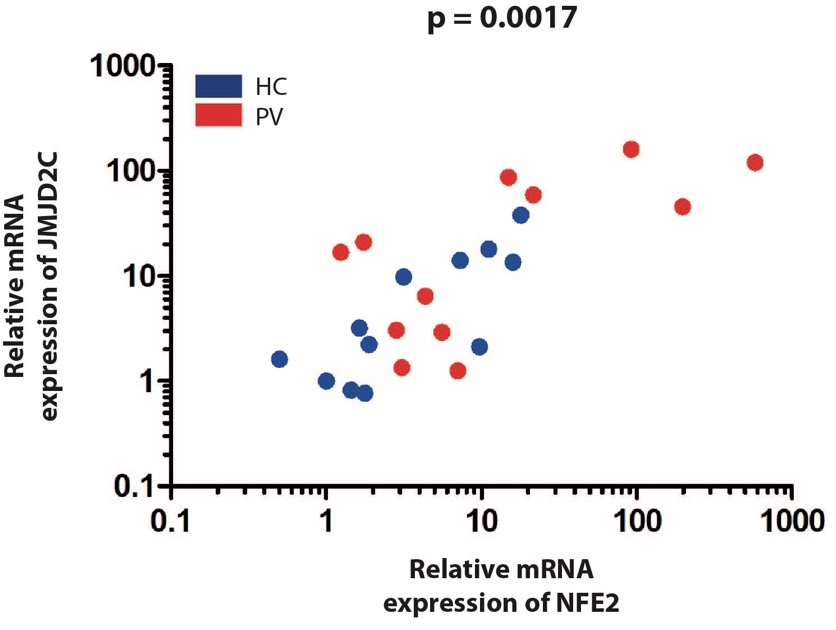
**

**Figure S2: Correlation of JMJD2C and NFE2 mRNA expression in PV patients.** *JMJD2C* and *NFE2* mRNA expression in PB granulocytes of n=12 healthy controls (blue) and n=12 PV patients (red) by RT-qPCR. Expression is normalized to *B2m* expression. Statistical significance was determined using the Pearson correlation coefficient.


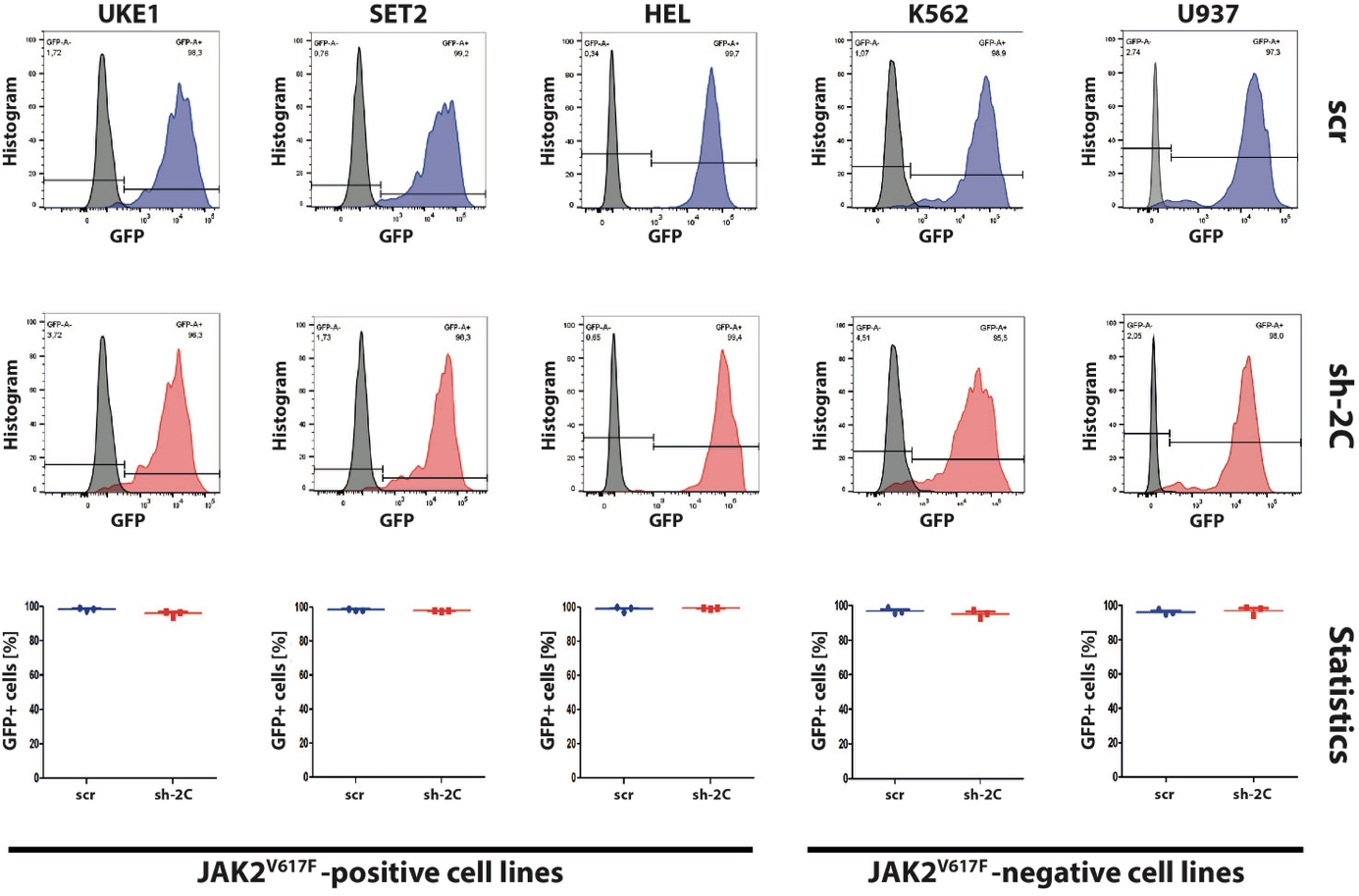


**Figure S3: Infection efficiency after lentiviral transduction.** FACS analysis of GFP^+^ cells in UKE1, SET2, HEL, K562 and U937 cells 5 days after lentiviral transduction with vectors carrying a scrambled control shRNA (top) or shRNA 2C #3 against *JMJD2C* (middle) as described in Figure 2. Representative FACS plots are shown (top, middle). Black (uninfected), blue (scr), red (sh-2C). Statistical representation of the GFP^+^ fraction (bottom). Data are represented as mean of three independent experiments.


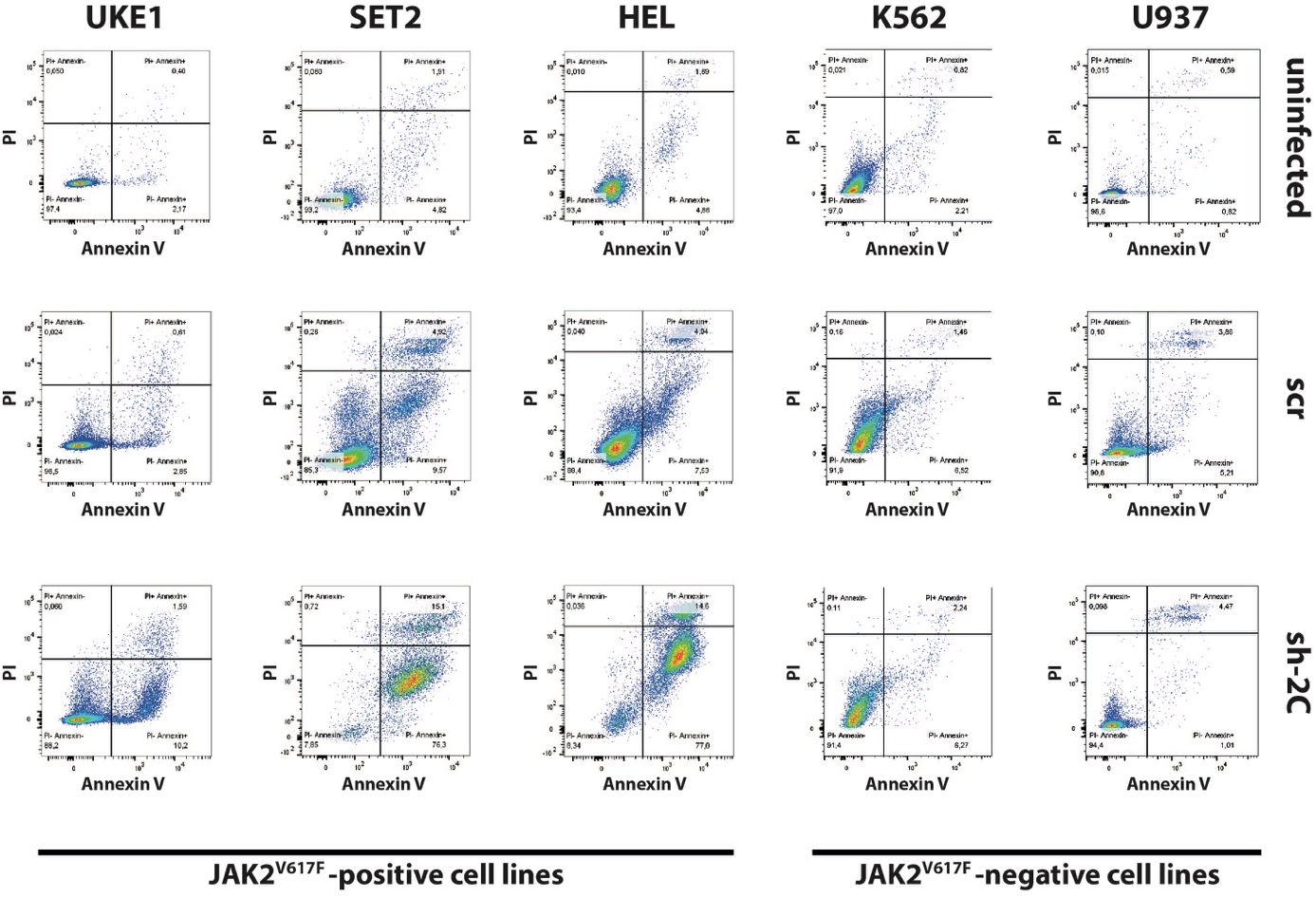


**Figure S4: AnnexinV/PI staining.** The proportions of apoptotic (AnnexinV+/PI-) and necrotic (AnnexinV+/PI+) cells were determined in uninfected UKE1, SET2, HEL, K562 and U937 cells (top). Apoptosis detection 9 days after lentiviral transduction with vectors carrying a scrambled control shRNA (middle) or shRNA 2C #3 against *JMJD2C* (bottom) as described in Figure 2. Representative FACS plots are shown.


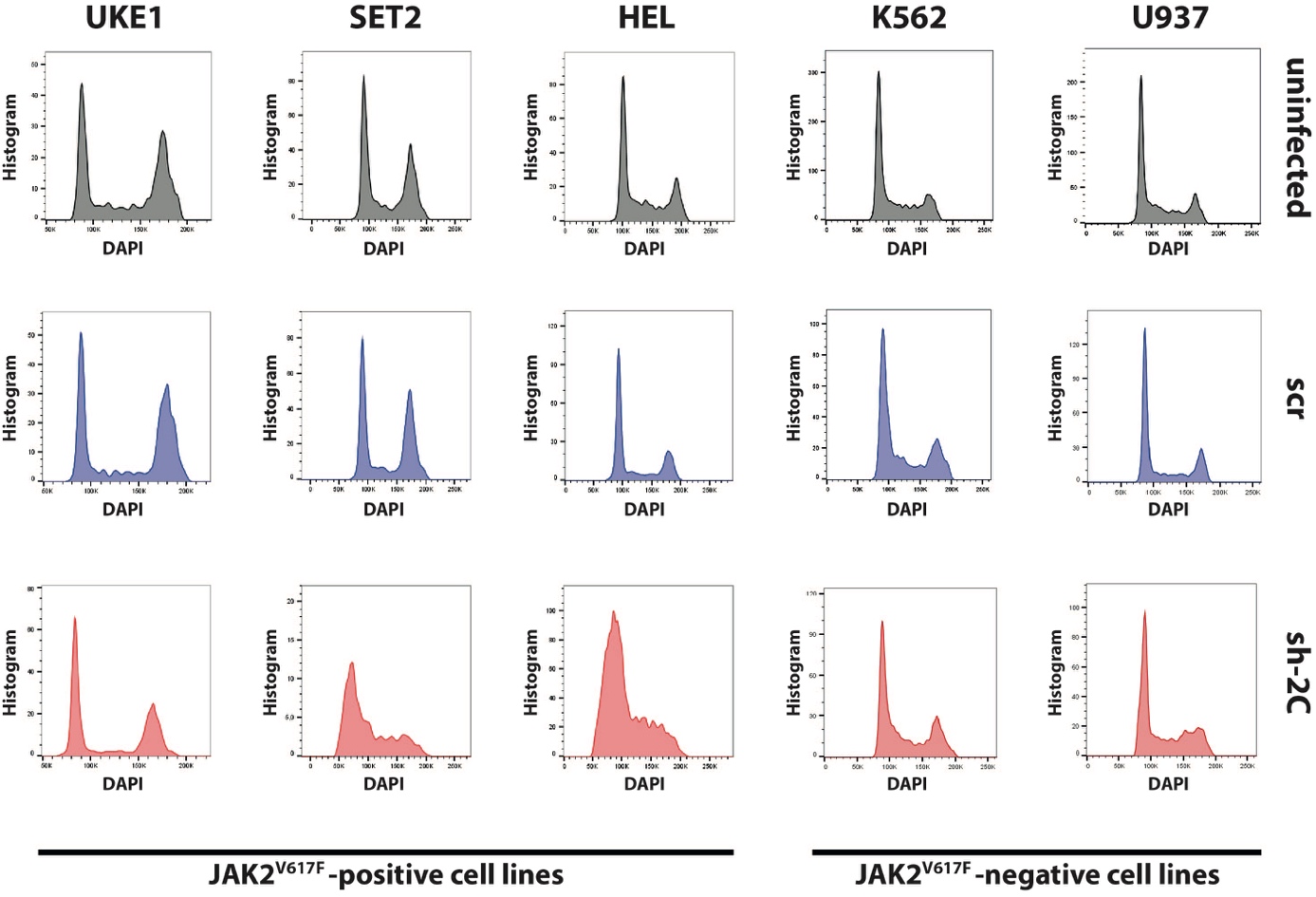


**Figure S5: Cell cycle analysis.** The proportions of cells in G1 (DAPI low), S (DAPI medium) and G2/M (DAPI high) phase were determined in uninfected UKE1, SET2, HEL, K562 and U937 cells (top). Cell cycle analysis 9 days after lentiviral transduction with vectors carrying a scrambled control shRNA (middle) or shRNA 2C #3 against *JMJD2C* (bottom) as described in Figure 2. Representative FACS plots are shown. Black (uninfected), blue (scr), red (sh-2C).

**
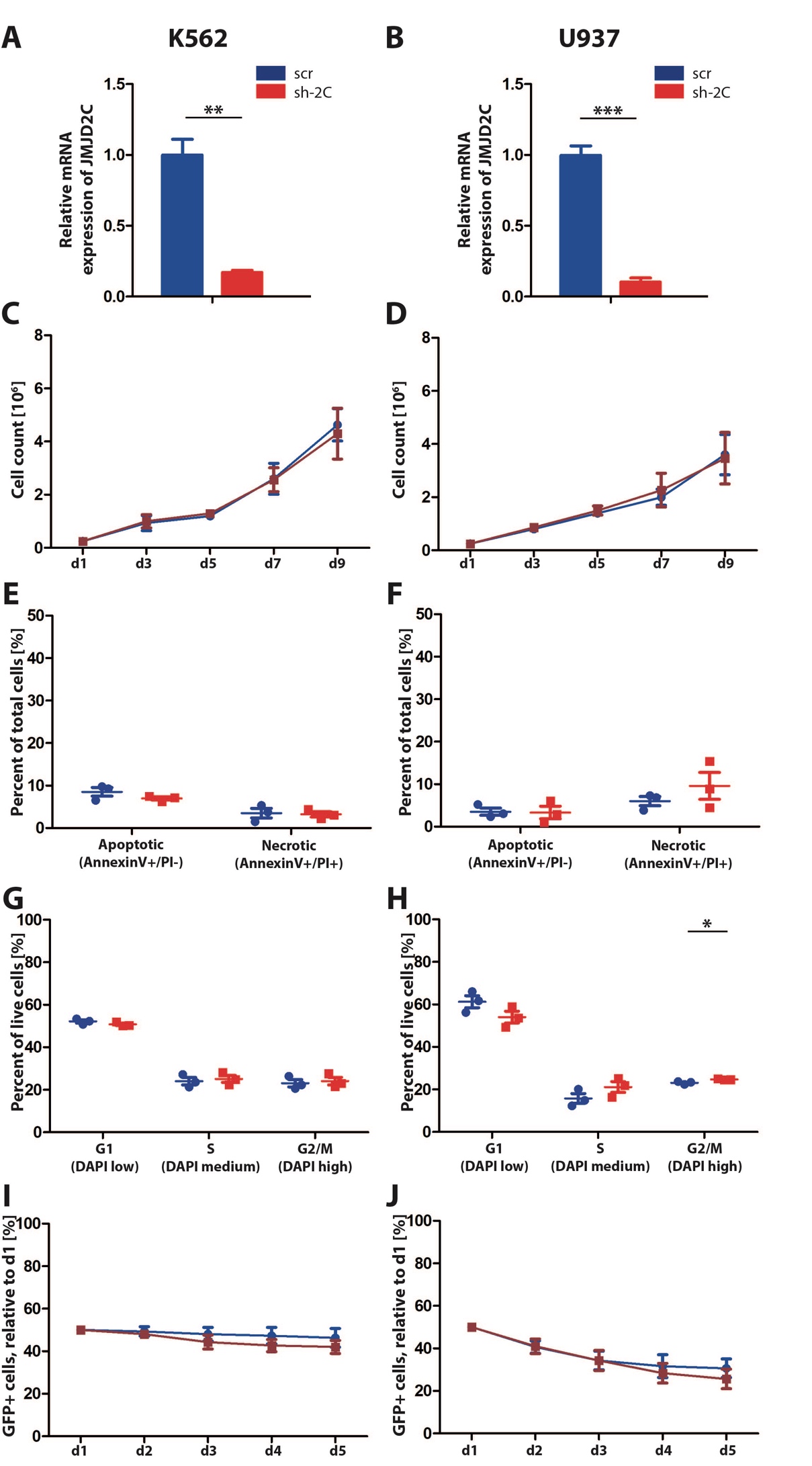
**

**Figure S6: JMJD2C is dispensable for proliferation of JAK2^V617F^-negative K652 and U937 cells. (A+B)** K562 **(A)** and U937 **(B)** cells were lentivirally transduced with vectors carrying shRNAs against *JMJD2C* (sh-2C) or a scrambled control (scr). Cells were harvested on day 5 for RNA isolation and subsequent analysis by RT-qPCR (n=3). **(C+D)** Proliferation of K562 **(C)** and U937 **(D)** cells after introduction of sh-2C #3 or the scr control shRNA. For each condition, 0.25 million cells were seeded and infected on day 1. Cells were counted every second day and used for final analysis on day 9. **(E+F)** Detection of apoptotic (AnnexinV+/PI-) and necrotic (AnnexinV+/PI+) cells on day 9 by FACS analysis in K562 **(E)** and U937 **(F)** cells. **(G+H)** Cell cycle analysis: Detection of cells in G1 (DAPI low), S (DAPI medium) and G2/M (DAPI high) phase on day 9 by FACS analysis in K562 **(G)** and U937 **(H)** cells. **(I+J)** Competitive growth of shRNA (sh-2C or scr) infected K562 **(I)** and U937 **(J)** cells. 0.1 million infected (GFP^+^) cells were seeded at a 1:1 ratio with uninfected cells on day 1. The proportion of GFP^+^ cells was determined daily. **(A-J)** Data are represented as mean +/- SEM of three independent experiments. *p<0.05, **p<0.01, ***p<0.01 by Student’s t test.

**
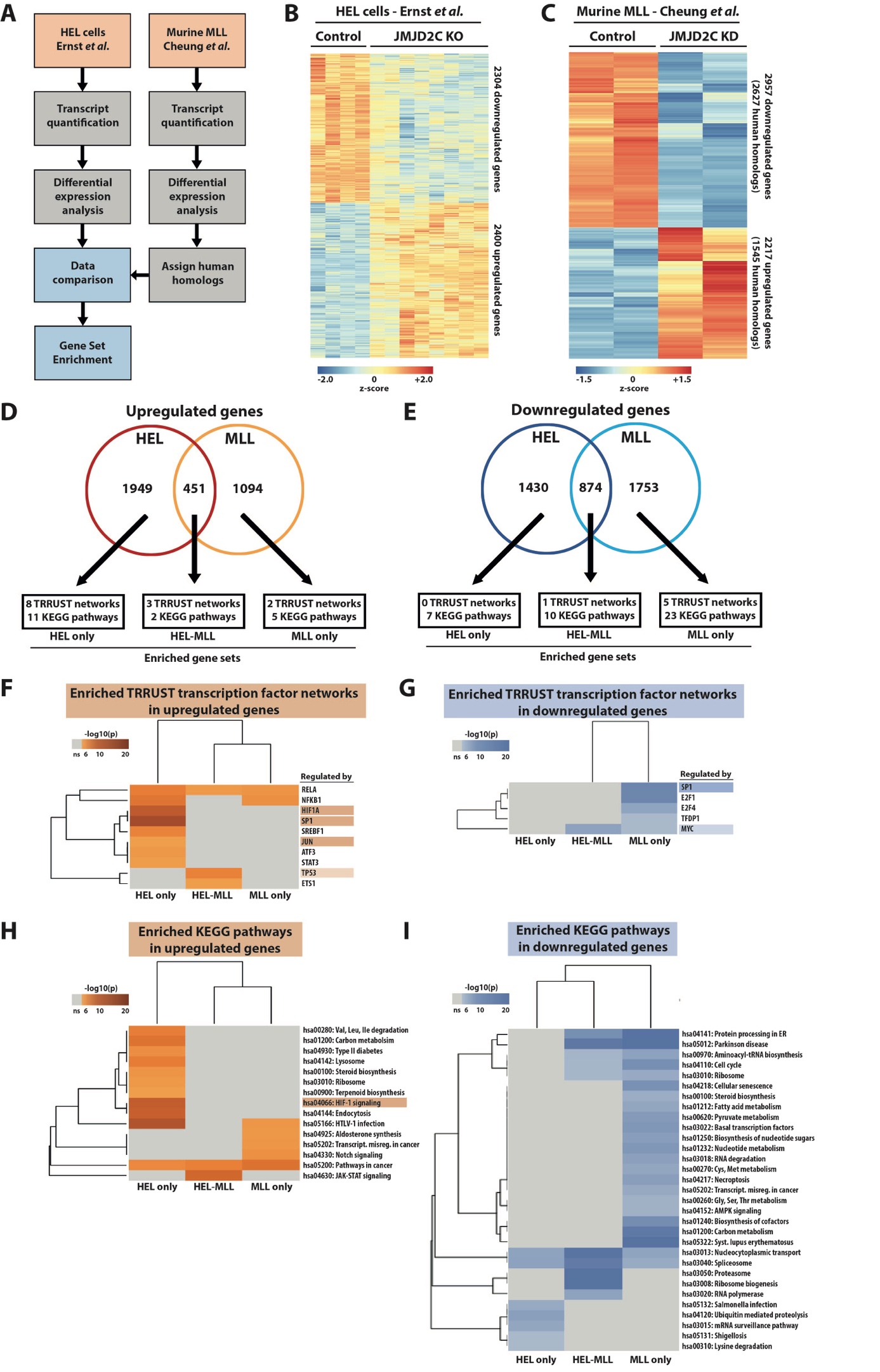
**

**Figure S7: Comparative RNA-seq analysis to determine JAK2^V617F^-specific effects caused by JMJD2C depletion in myeloid cells. (A- I)** RNA-seq datasets published by Ernst *et al.* (1) and Cheung *et al.* (2) were obtained from the Sequence Read Archive. The following conditions from Ernst et al. were analyzed: JMJD2C KO in HEL cells (n=8) compared to control HEL cells (n=4). The following conditions from Cheung *et al.* were analyzed: JMJD2C KD in murine MLL cells (n=2) compared to control murine MLL cells (n=2). KO: knockout, KD: knockdown. **(A)** Workflow of the RNA-seq data analysis. Transcript quantification was performed with Salmon while differential expression analysis was conducted with DESeq2. For the Cheung *et al.* dataset, which is derived from murine bone marrow cells, human homologs were assigned to the murine genes using HomoloGene. Gene Set Enrichment of TRRUST transcription factor networks and KEGG pathways was performed using Metascape. **(B)** Heatmap of genes differentially expressed in JMD2C KO HEL cells (n=8) compared to WT HEL cells (n=4), Padj < 0.05. (**C)** Heatmap of genes differentially expressed in JMD2C KD MLL cells (n=2) compared to WT MLL cells (n=2), Padj < 0.05. Differentially expressed genes were assigned to their human homologs (in brackets, see (A)). (**D-I)** For subsequent analyses of the Cheung *et al.* dataset, human homologs of the differentially expressed genes were used. **(D+E)** Venn Diagrams depicting genes upregulated and downregulated compared to control cells in the individual data sets as well as genes altered by JMJD2C depletion in both datasets (overlap). Genes were assigned to following groups: upregulated (**D**) or downregulated (**E**) only in HEL cells (HEL-only), only in MLL cells (MLL-only) or in both HEL and MLL cells (HEL-MLL). These groups of genes were subsequently used for Gene Set Enrichment analysis. The number of significantly enriched TRRUST networks and KEGG pathways are depicted for each group. (**F-H)** Enrichment analysis of TRRUST networks (**F+G**) and KEGG pathways (**H+I**) in upregulated (**F+H**) and downregulated (**G+I**) genes. Selected TRRUST networks are highlighted, see text. Cutoff for significant enrichment: p< 0.00001.

**Materials and Methods**

Chromatin immunoprecipitation

Chromatin immunoprecipitations (ChIP) were performed as previously described (3). Antibodies are listed in **Table S1**. For the ChIP polymerase chain reaction (PCR), input DNA was diluted 1:10. Primers used for DNA amplification are listed in **Table S2**.

RT-qPCR and RT-PCR

RNA isolation and reverse transcription were performed as previously described (4,5). cDNA from PV patients, hNFE2tg mice and cell lines (CB3, UKE1, SET2, HEL, K562, U937) was used for quantitative RT-PCR (RT-qPCR). The expression of *Jmjd2c* (Thermo Fisher Scientific, Waltham, Massachusetts, USA, Mm01263760m1, Hs00909577m1) and *NFE2* (Thermo Fisher Scientific, Assay on Demand) was tested. β-2-microglobulin (*B2m*) was used as housekeeping gene (Thermo Fisher Scientific, Assay on Demand). Data were analyzed using the ΔΔCT method (6).

Lentiviral transduction

Modified pLeGO-iG-U6 vectors were used for lentiviral transduction as previously described (7). The pLeGO-iG-hU6-hNFE2 construct was used for overexpression of human NFE2 in CB3 cells. To study JMJD2C depletion, *Jmjd2c* shRNAs and a scrambled control shRNA were introduced into UKE1, SET2, HEL, K562 and U937 cells using the pLeGO-iG-hU6-sh-2C-#1-3 and pLeGO-iG-hU6-sh-scr constructs. shRNA sequences and the plasmids used are listed in **Table S2** and **S3**.

Luciferase assays

Luciferase assays were performed as previously described (8). The plasmids used for these assays are listed in **Table S3**. To generate a reporter plasmid containing the *JMJD2C* promoter element, we amplified the *JMJD2C* promoter region ranging from -2228 to + 552 bp relative to the transcription start site (TSS) by PCR using gDNA extracted from HEL cells. The PCR fragment was subsequently cloned into the pGL4.10 luciferase reporter vector (Promega, Madison, Wisconsin, USA). Employing the GeneArt site-directed mutagenesis system (Thermo Fisher Scientific), the +260 bp NFE2-binding site within the *JMJD2C* promoter was mutated from “TGACTCA” to “ATTCATT”. Primers used for cloning are listed in **Table S2**.

Study approval

Peripheral blood (PB) samples were obtained from patients with polycythemia vera (PV) meeting the World Health Organization (WHO) criteria for diagnosis (9). Buffy coats of healthy blood donors were used as controls. The study protocol was approved by the Ethics Committee at the University Center Freiburg (Application Number 558/15). Informed consent was obtained from all patients included in the study.

Granulocyte isolation

As previously described, granulocytes were isolated from PB samples by dextran sedimentation and Ficoll gradient centrifugation (10).

Immunoblotting

Antibodies used for immunoblotting, performed as previously described (4,5), are listed in **Table S1**. Immune complexes were detected by chemiluminescence (PerkinElmer, Waltham, Massachusetts, USA, NEL103001EA). Densitometric analysis was performed using the National Institutes of Health ImageJ software (11).

Animal housing and protection

Experiments were approved by the Environment and Consumer Protection Agency of the State of Baden-Württemberg, Germany (G-07/59). Animal housing and welfare measures were recently described in detail (5).

Cell cycle analysis

Cells were harvested and fixed in 2 % formalin for 15 minutes followed by permeabilization for 30 minutes in PBS containing 0.1 % TritonX-100 (Sigma-Aldrich, St. Louis, Missouri, USA, T8787). DAPI staining was performed for 30 minutes at room temperature protected from light in a PBS based solution containing 1 μg/ml DAPI (BioLegend, San Diego, California, USA, 422801). The DAPI signal was detected on a BD FACS Fortessa using a 405 nm laser and a 450/50 nm filter.

Apoptosis detection

Apoptotic and necrotic cells were stained using the Pacific Blue^TM^ Annexin V Apoptosis Detection Kit with PI (BioLegend, 64098) according to manufacturer’s instructions and detected on a BD FACS Fortessa.

RNA-seq analysis

RNA-seq datasets SRP375293 and ERP009549 were obtained from the Sequence Read Archive (12). The Trim Galore! (13) and Salmon (14) tools on the Galaxy online platform (usegalaxy.eu, (15)) were used for read trimming and transcript quantification. Reference genomes for transcript quantification were obtained from the GENCODE project (16). Raw counts were imported with tximport (17) and differential expression analysis was performed with DESeq2 (18). After differential expression analysis, human homologs were assigned using HomoloGene (NCBI). Differentially expressed genes from both datasets were compared and Gene Set Enrichment of KEGG pathways (19) and TRRUST (20) transcription factor networks was analyzed using the online tool Metascape (21).

Statistics

GraphPad PRISM version 6 (GraphPad Software, San Diego, California, USA) was used for statistical calculations. Comparisons between two groups were conducted using either the Student’s t test or the Mann-Whitney U test, as appropriate, while survival analyses were performed using the Log-rank (Mantel Cox) test. Statistical significance of correlation analyses was determined using the Pearson correlation coefficient.

**Table S1: List of antibodies**

| **Target** | **Catalog #** | **Company** | **Application** | **Figure** |
| --- | --- | --- | --- | --- |
| Anti-NFE2 | Sc-291 | Santa Cruz, Dallas, Texas, USA | ChIP | 1 |
| Anti-IgG | 27295 | Cell Signaling, Cambridge, UK | ChIP | 1 |
| Anti-JMJD2C | AB85454 | Abcam, Cambridge, UK | WB | 1/2 |
| Anti-GAPDH | G8795 | Sigma-Aldrich | WB | 1 |
| Anti-β-ACTIN | A5441 | Sigma-Aldrich | WB | 2 |
| Anti-H3K9me3 | 61014 | Active motif, Carlsbad, California, USA | WB | 2 |
| Anti-H3K27me3 | 61018 | Active motif | WB | 2 |
| Anti-H3K36me3 | C15410192 | Diagenode, Denville, New Jersey, USA | WB | 2 |
| Anti-H3 | 9715S | Cell Signaling | WB | 2 |
| Anti-Rabbit IgG HRP | NA934V | GE Healthcare, Chicago, Illinois, USA | WB | 1/2 |
| Anti-Mouse IgG HRP | NA931V | GE Healthcare | WB | 1/2 |

**Table S2: List of primers**

| **Name** | **Sequence 5’ to 3’** | **Application** | **Figure** |
| --- | --- | --- | --- |
| 2C +260 bp fw | CCACAGCGCGGAAGTTGAGC | ChIP-PCR | 1 |
| 2C +260 bp rv | TGGGAGGCAGCGGCGAAG | ChIP-PCR | 1 |
| 2C +4.6 kb fw | CTGCACTGATCAACTCGTTG | ChIP-PCR | 1 |
| 2C +4.6 kb rv | GAACACATGGACACAGGGAG | ChIP-PCR | 1 |
| Myo locus fw | AGGGGCTGCTGAGAAATGAAAAC | ChIP-PCR | 1 |
| Myo locus rv | ATATAGCCAACGCCACAGAAACCT | ChIP-PCR | 1 |
| JMJD2C fmut13 | GTAAGCCCACGATTCATTCCAAGTGCGGGCC | SDM | 1 |
| JMJD2C rmut17 | CCGCACTTGGAATGAATCGTGGGCTTACAAACAG | SDM | 1 |
| sh_scr_sense | GGGTGAACTCACGTCAGAA | shRNA | 2 |
| sh_scr_anti | TTCTGACGTGAGTTCACCC | shRNA | 2 |
| sh_2C_#1_sense | TAAATAAAACACATCTCTGGTA **(*1)** | shRNA | 2 |
| sh_2C_#1_anti | TACCAGAGATGTGTTTTATTTA **(*1)** | shRNA | 2 |
| sh_2C_#2_sense | GCAGGTGGAGCAGAATTTATC **(*2)** | shRNA | 2 |
| sh_2C_#2_anti | GATAAATTCTGCTCCACCTGC **(*2)** | shRNA | 2 |
| sh_2C_#3_sense | AGATAGCAGCAATGAAGAA **(*3)** | shRNA | 2 |
| sh_2C_#3_anti | TTCTTCATTGCTGCTATCT **(*3)** | shRNA | 2 |

***1** Created with splashRNA (22). ***2** Sequence from Huang *et al.* (23). ***3** Sequence from Pedersen *et al.* (24).

**Table S3: List of plasmids**

| **Name** | **Backbone** | **Insert** | **Application** | **Figure** |
| --- | --- | --- | --- | --- |
| pLeGO-iG-hU6-empty | pLego-iG-hU6 | empty | Control / Cloning | 1 |
| pLego-iG-hU6-hNFE2-wt | pLego-iG-hU6 | hNFE2-wt | Overexpression | 1 |
| pLeGO-iG-hU6-sh-scr | pLego-iG-hU6 | sh-scr | Knockdown | 2 |
| pLeGO-iG-hU6-sh-2C-#1 | pLego-iG-hU6 | sh-2C-#1 | Knockdown | 2 |
| pLeGO-iG-hU6-sh-2C-#2 | pLego-iG-hU6 | sh-2C-#2 | Knockdown | 2 |
| pLeGO-iG-hU6-sh-2C-#3 | pLego-iG-hU6 | sh-2C-#3 | Knockdown | 2 |
| pCMV-dR8.47 | pCMV | gag/pol | Lentiviral packaging | 1/2 |
| pMD2.G-VSV-G | pMD2.G | VSV-G | Lentiviral envelope | 1/2 |
| pCMV6XL4 | pCMV6XL4 | empty | Luciferase Assay | 1 |
| pCMV6XL4-MafG | pCMV6XL4 | MafG | Luciferase Assay | 1 |
| pRc/CMV | pRc/CMV | empty | Luciferase Assay | 1 |
| pRc/CMV-NFE2 | pRc/CMV | NFE2 | Luciferase Assay | 1 |
| pRL-TK Renilla | pRL | TK Renilla | Luciferase Assay | 1 |
| pGL4.10 | pGL4.10 | empty | Luciferase Assay | 1 |
| pGL4.10_2C_promoter | pGL4.10 | 2C_promotor | Luciferase Assay | 1 |

**References**

1. Ernst P, Schnöder TM, Huber N, Perner F, Jayavelu AK, Eifert T, et al. Histone demethylase KDM4C is a functional dependency in JAK2-mutated neoplasms. Leukemia. 2022 Jul;36(7):1843–9.

2. Cheung N, Fung TK, Zeisig BB, Holmes K, Rane JK, Mowen KA, et al. Targeting Aberrant Epigenetic Networks Mediated by PRMT1 and KDM4C in Acute Myeloid Leukemia. Cancer Cell. 2016 Jan 11;29(1):32–48.

3. Wang W, Schwemmers S, Hexner EO, Pahl HL. AML1 is overexpressed in patients with myeloproliferative neoplasms and mediates JAK2V617F-independent overexpression of NF-E2. Blood. 2010 Jul 15;116(2):254–66.

4. Peeken JC, Jutzi JS, Wehrle J, Koellerer C, Staehle HF, Becker H, et al. Epigenetic regulation of NFE2 overexpression in myeloproliferative neoplasms. Blood. 2018 May 3;131(18):2065–73.

5. Staehle HF, Heinemann J, Gruender A, Omlor AM, Pahl HL, Jutzi JS. Jmjd1c is dispensable for healthy adult hematopoiesis and Jak2V617F-driven myeloproliferative disease initiation in mice. PLOS ONE. 2020 Apr 2;15(2):e0228362.

6. Livak KJ, Schmittgen TD. Analysis of relative gene expression data using real-time quantitative PCR and the 2(-Delta Delta C(T)) Method. Methods San Diego Calif. 2001 Dec;25(4):402–8.

7. Roelz R, Pilz IH, Mutschler M, Pahl HL. Of mice and men: human RNA polymerase III promoter U6 is more efficient than its murine homologue for shRNA expression from a lentiviral vector in both human and murine progenitor cells. Exp Hematol. 2010 Sep;38(9):792–7.

8. Wehrle J, Seeger TS, Schwemmers S, Pfeifer D, Bulashevska A, Pahl HL. Transcription factor nuclear factor erythroid-2 mediates expression of the cytokine interleukin 8, a known predictor of inferior outcome in patients with myeloproliferative neoplasms. Haematologica. 2013 Jul;98(7):1073–80.

9. Arber DA, Orazi A, Hasserjian R, Thiele J, Borowitz MJ, Le Beau MM, et al. The 2016 revision to the World Health Organization classification of myeloid neoplasms and acute leukemia. Blood. 2016 May 19;127(20):2391–405.

10. Temerinac S, Klippel S, Strunck E, Röder S, Lübbert M, Lange W, et al. Cloning of PRV-1, a novel member of the uPAR receptor superfamily, which is overexpressed in polycythemia rubra vera. Blood. 2000 Apr 15;95(8):2569–76.

11. Schneider CA, Rasband WS, Eliceiri KW. NIH Image to ImageJ: 25 years of image analysis. Nat Methods. 2012 Jul;9(7):671–5.

12. Leinonen R, Sugawara H, Shumway M. The Sequence Read Archive. Nucleic Acids Res. 2011 Jan;39(Database issue):D19–21.

13. Krueger F. Babraham Bioinformatics - Trim Galore! [Internet]. [cited 2022 Dec 4]. Available from: https://www.bioinformatics.babraham.ac.uk/projects/trim_galore/

14. Patro R, Duggal G, Love MI, Irizarry RA, Kingsford C. Salmon provides fast and bias-aware quantification of transcript expression. Nat Methods. 2017 Apr;14(4):417–9.

15. Afgan E, Baker D, Batut B, van den Beek M, Bouvier D, Čech M, et al. The Galaxy platform for accessible, reproducible and collaborative biomedical analyses: 2018 update. Nucleic Acids Res. 2018 Jul 2;46(W1):W537–44.

16. Frankish A, Diekhans M, Ferreira AM, Johnson R, Jungreis I, Loveland J, et al. GENCODE reference annotation for the human and mouse genomes. Nucleic Acids Res. 2019 Jan 8;47(D1):D766–73.

17. Soneson C, Love MI, Robinson MD. Differential analyses for RNA-seq: transcript-level estimates improve gene-level inferences [Internet]. F1000Research; 2016 [cited 2022 Dec 4]. Available from: https://f1000research.com/articles/4-1521

18. Love MI, Huber W, Anders S. Moderated estimation of fold change and dispersion for RNA-seq data with DESeq2. Genome Biol. 2014 Dec 5;15(12):550.

19. Kanehisa M, Goto S. KEGG: kyoto encyclopedia of genes and genomes. Nucleic Acids Res. 2000 Jan 1;28(1):27–30.

20. Han H, Cho JW, Lee S, Yun A, Kim H, Bae D, et al. TRRUST v2: an expanded reference database of human and mouse transcriptional regulatory interactions. Nucleic Acids Res. 2018 Jan 4;46(D1):D380–6.

21. Zhou Y, Zhou B, Pache L, Chang M, Khodabakhshi AH, Tanaseichuk O, et al. Metascape provides a biologist-oriented resource for the analysis of systems-level datasets. Nat Commun. 2019 Apr 3;10(1):1523.

22. Pelossof R, Fairchild L, Huang CH, Widmer C, Sreedharan VT, Sinha N, u. a. Prediction of potent shRNAs with a sequential classification algorithm. Nat Biotechnol. Apr 2017;35(4):350–3.

23. Huang B, Wang B, Yuk-Wai Lee W, Pong U K, Leung KT, Li X, u. a. KDM3A and KDM4C Regulate Mesenchymal Stromal Cell Senescence and Bone Aging via Condensin-mediated Heterochromatin Reorganization. iScience. 22. Nov 2019;21:375–90.

24. Pedersen MT, Agger K, Laugesen A, Johansen JV, Cloos PAC, Christensen J, u. a. The demethylase JMJD2C localizes to H3K4me3-positive transcription start sites and is dispensable for embryonic development. Mol Cell Biol. Mar 2014;34(6):1031–45.
